# Supplementary material for: The PYY/Y2R-Deficient Mouse Responds Normally to High-Fat Diet and Gastric Bypass Surgery
Source: Nutrients. 2019 Mar 10;11(3):585. doi: 10.3390/nu11030585 (PMC6471341; doi:10.3390/nu11030585)
Supplement: Supplementary file 1 [file nutrients-11-00585-s001.pdf]

nutrients

MDPI

Supplementary Table S1. Fatsing and postprandial plasma concentrations (ng/mL) of hormones in WT and Y2RKO mice on high-fat diet.

	Fasting		Postprandial	
Peptide	WT	Y2RKO	WT	Y2RKO
PYY	107.2 ± 8.1	136.5 ± 7.7	166.5 ± 13.8	145.8 ± 28.7
GLP-2	2.42 ± 0.21	3.07 ± 0.14	2.49 ± 0.19	2.93 ± 0.60
Resistin	19549 ± 1597	17294 ± 2564	23162 ± 1861	18148 ± 2017
GIP	266.4 ± 33.9	343.1 ± 101.7	365.9 ± 82.2	233.0 ± 60.1
Glucagon	68.3 ± 7.4	58.8 ± 8.8	90.5 ± 8.2	71.1 ± 7.2
Leptin	15525 ± 2057	12567 ± 2546	18390 ± 2346	15187 ± 3604

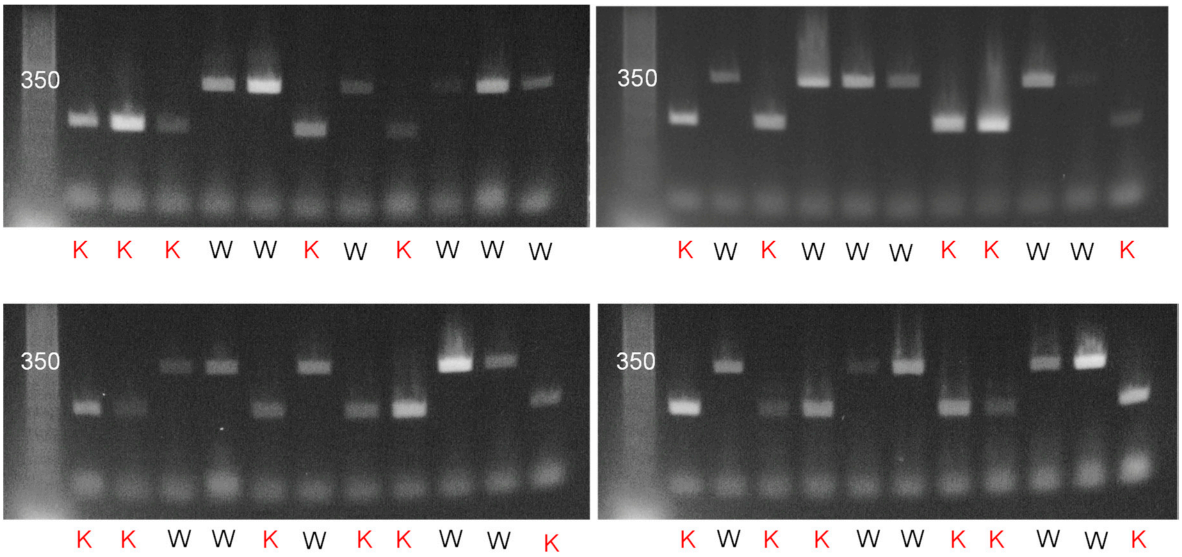

Supplementary Figure S1. Genotyping of the 22 WT (W) and 22 Y2R$^{-/-}$/GLP1R$^{-/-}$ double-knockout mice used for the final analysis. Tail biopsies, taken at time of tissue harvest, were incubated at 95 °C in lysis solution for 1hr. Neutralization buffer was added, and tails were stored at -20 °C until time of genotyping. The PCR sample consisted of a 25μL volume containing 1μL of each of three primers (11982_3: TTGATCTCACTCATTGTGGAGC; 11982_4: CATCAATTGATGAAGATACAGGC; 11982_7: TCTACAGTTTGATTCTCATCTGCC). Each reaction also contained AmpliTaq Gold 360 Master Mix and 360 GC Enhancer (Applied Biosystems by Thermo Fisher Scientific). The following PCR conditions were applied: 5 min, 95 °C initial denaturation; 30 s, 95 °C cyclic denaturation; 30 s, 60 °C cyclic annealing; 1 min, 72 °C cyclic elongation for a total of 35 cycles, followed by a 10 min 72 °C elongation step. PCR amplification products were analyzed by agarose gel electrophoresis. KO band are detected at 206 base pairs and WT band is detected at 357 base pairs.

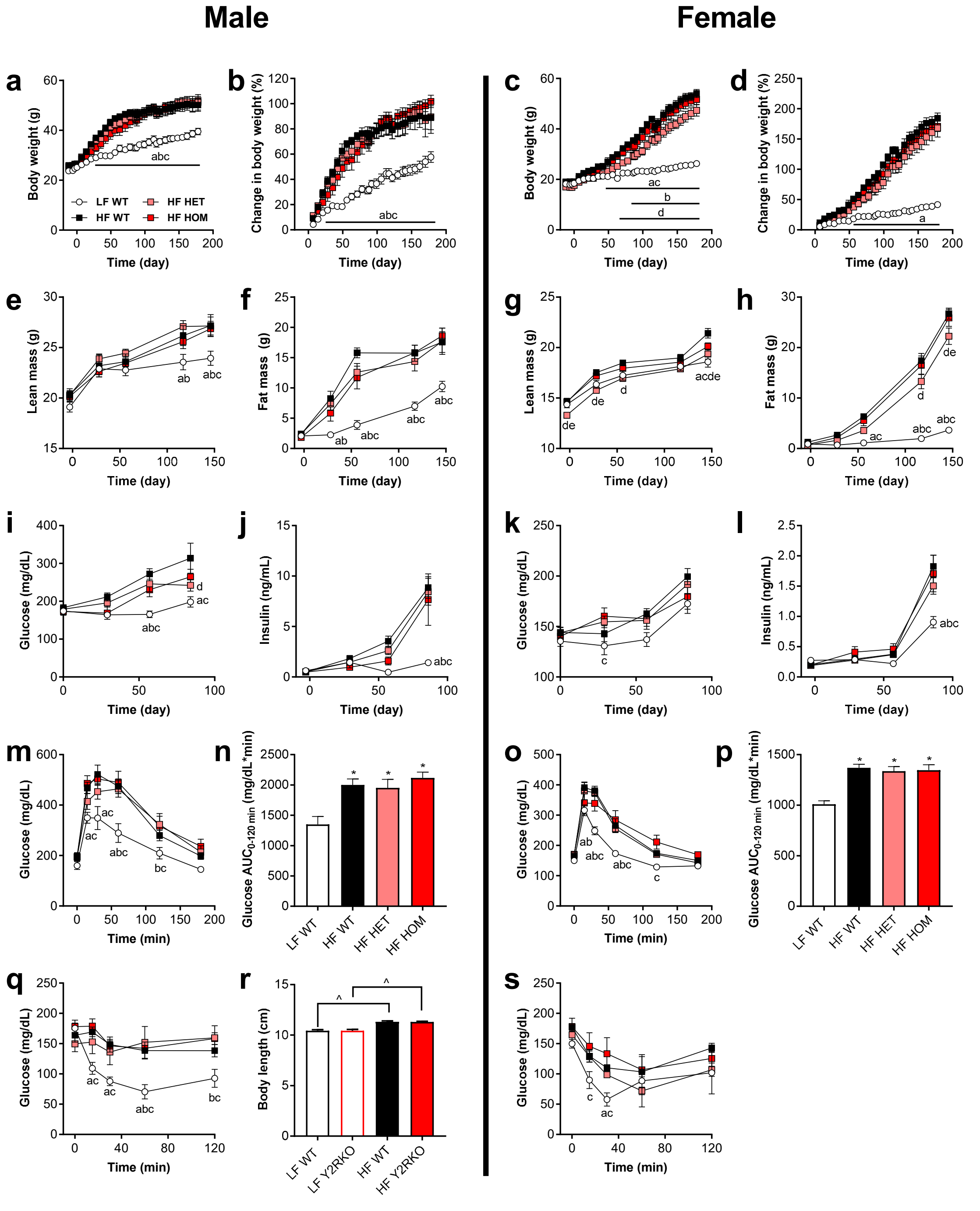

Supplementary Figure S2. Physiological parameters of male and female wildtype (WT), heterozygous (HET) and homozygous Y2RKO mice on 60% high-fat (HF) diet, compared to WT mice on 10% low-fat (LF) diet. (**a**–**d**) Absolute and percent change in body weight. (**e**–**h**) Total lean and fat mass. **(i**–**l)** Fasting blood glucose and fasting plasma insulin. **(m**–**p)** Intraperitoneal glucose tolerance test (1.5 g/kg) and associated area under the curve (AUC). **(q, s)** Intraperitoneal insulin tolerance test (1.0 U/kg). n = 7–13 for all groups. $^{a}p \leq 0.05$ LF WT vs HF WT, $^{b}p \leq 0.05$ LF WT vs HF HET, $^{c}p \leq 0.05$ LF WT vs. HF HOM, $^{d}p \leq 0.05$ HF WT vs. HF HET, $^{e}p \leq 0.05$ HF HET vs. HF HOM. **r:** Terminal body length in a different cohort of male mice, the same cohort as that shown in Figure 1 (n = 9–10/group). ^ $p \leq 0.0001$)

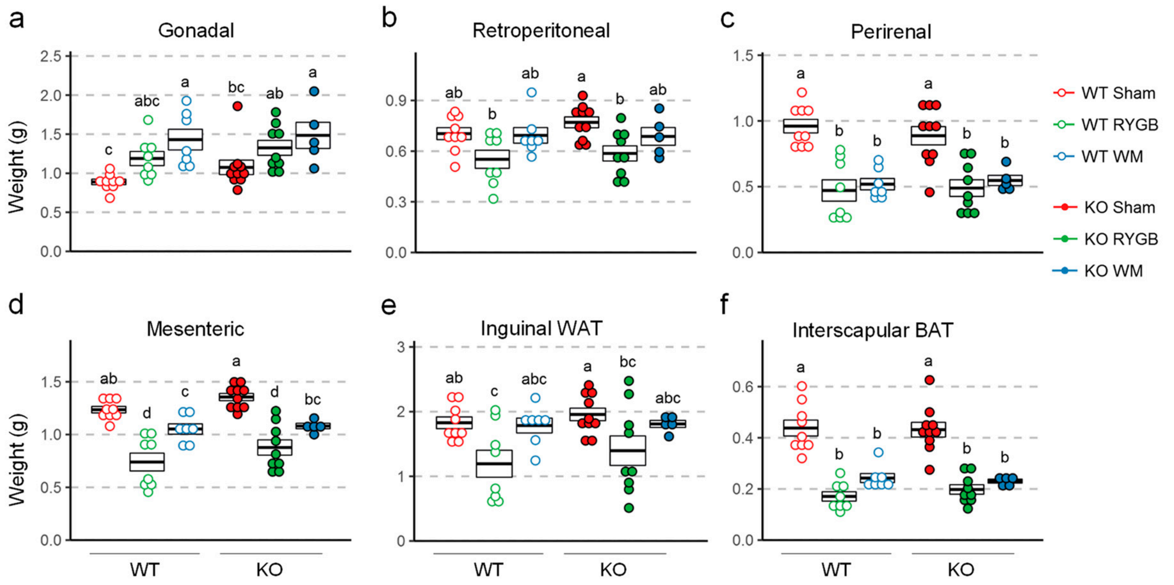

Supplementary Figure S3. Effect of RYGB, sham surgery, or weight matching to RYGB by caloric restriction (WM) on weight of gonadal **(a)**, retroperitoneal **(b)**, perirenal **(c)**, mesenteric **(d)**, and inguinal white fat **(e)**, as well as interscapular brown fat pads **(f)** in Y2RKO and WT mice at termination of the experiment. Data are expressed as individual data points over a box showing means ± SEM. Data that do not share the same letters are significantly different from each other ($p < 0.05$, pairwise t-tests with Benjamini-Hochberg correction, FDR = 0.05).

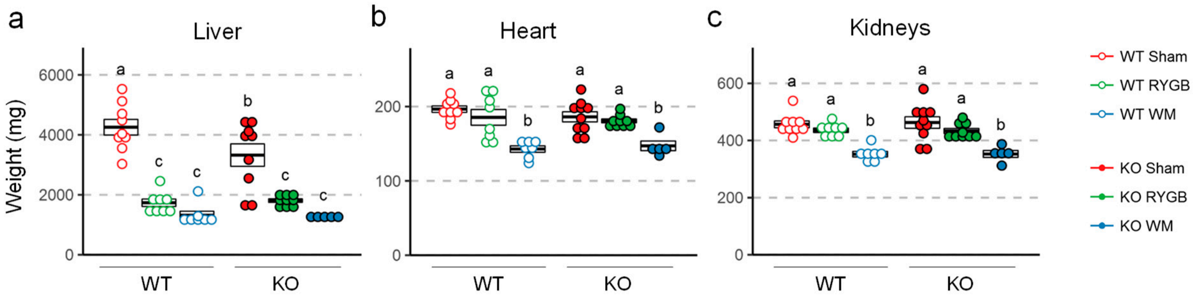

Supplementary Figure S4. Effect of RYGB, sham surgery, or weight matching to RYGB by caloric restriction (WM) on liver **(a)**, heart **(b)**, and kidney **(c)** weights in Y2RKO and WT mice at termination of the experiment. Data are expressed as individual data points over a box showing means ± SEM. Data that do not share the same letters are significantly different from each other ($p < 0.05$, pairwise *t*-tests with Benjamini-Hochberg correction, FDR = 0.05).

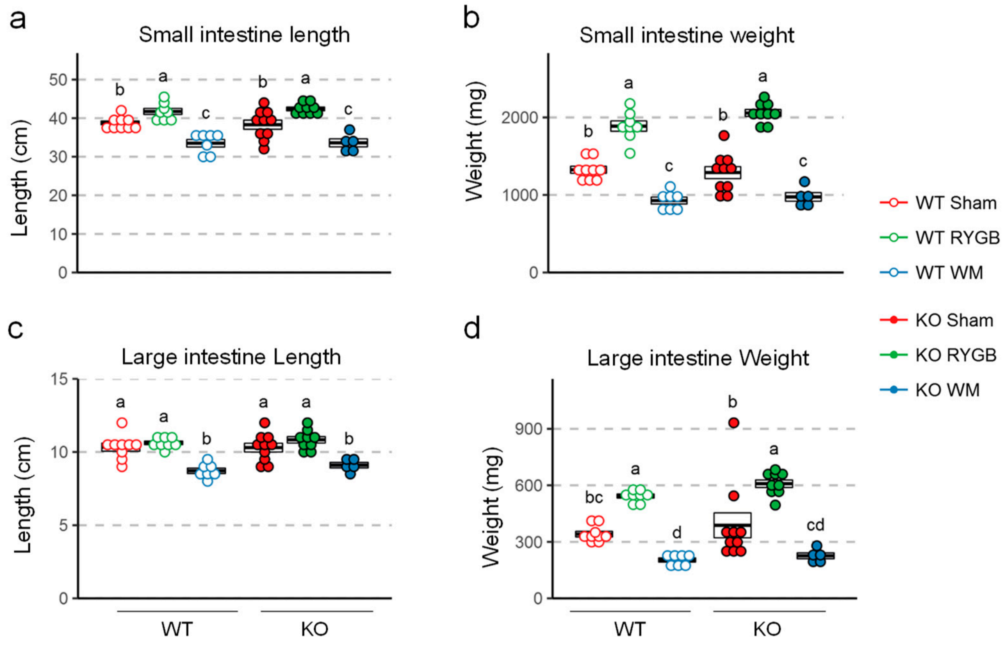

Supplementary Figure S5. Effect of RYGB, sham surgery, or weight matching to RYGB by caloric restriction (WM) on length (**a**,**c**) and weight (**b**,**d**) of small and large intestine, respectively in Y2RKO and WT mice at termination of the experiment. Data are expressed as individual data points over a box showing means ± SEM. Data that do not share the same letters are significantly different from each other ($p < 0.05$, pairwise t-tests with Benjamini-Hochberg correction, FDR = 0.05).

© 2019 by the authors. Submitted for possible open access publication under the terms and conditions of the Creative Commons Attribution (CC BY) license (http://creativecommons.org/licenses/by/4.0/).
